# Prediction intervals for future BMI values of individual children - a non-parametric approach by quantile boosting

**DOI:** 10.1186/1471-2288-12-6

**Published:** 2012-01-25

**Authors:** Andreas Mayr, Torsten Hothorn, Nora Fenske

**Affiliations:** 1 Institut für Medizininformatik, Biometrie und Epidemiologie, Friedrich-Alexander-Universität Erlangen-Nürnberg, Germany; 2 Institut für Statistik, Ludwig-Maximilians-Universität München, Germany

## Abstract

**Background:**

The construction of prediction intervals (PIs) for future body mass index (BMI) values of individual children based on a recent German birth cohort study with *n *= 2007 children is problematic for standard parametric approaches, as the BMI distribution in childhood is typically skewed depending on age.

**Methods:**

We avoid distributional assumptions by directly modelling the borders of PIs by additive quantile regression, estimated by boosting. We point out the concept of conditional coverage to prove the accuracy of PIs. As conditional coverage can hardly be evaluated in practical applications, we conduct a simulation study before fitting child- and covariate-specific PIs for future BMI values and BMI patterns for the present data.

**Results:**

The results of our simulation study suggest that PIs fitted by quantile boosting cover future observations with the predefined coverage probability and outperform the benchmark approach. For the prediction of future BMI values, quantile boosting automatically selects informative covariates and adapts to the age-specific skewness of the BMI distribution. The lengths of the estimated PIs are child-specific and increase, as expected, with the age of the child.

**Conclusions:**

Quantile boosting is a promising approach to construct PIs with correct conditional coverage in a non-parametric way. It is in particular suitable for the prediction of BMI patterns depending on covariates, since it provides an interpretable predictor structure, inherent variable selection properties and can even account for longitudinal data structures.

## Background

Childhood obesity is more and more becoming a problem of epidemic dimensions in modern societies [1,2]. The body mass index (BMI) has proved to be a reliable measure to assess childhood obesity and can also be seen as an indicator for obesity in adulthood [3,4]. Therefore, the prediction of future BMI values for individual children may be used as a warning bell for clinicians, parents and children. Predicting future BMI values raises awareness for problems to come - as long as they are still avoidable - and can thus lower the risk of later obesity.

In this setting, we focus on obtaining reliable predictions for future BMI values of children. Prediction intervals (PIs) offer information on the expected variability by providing not only a point prediction but a covariate-specific interval which covers the future BMI for this individual child with high probability. We construct child-specific prediction intervals for the LISA study, a recent German birth cohort study with 2007 children [5]. Data include up to ten BMI values per child from birth until the age of 10, as well as variables that are discussed to be potential early childhood risk factors for later obesity, such as breastfeeding, maternal BMI gain and smoking during pregnancy, parental overweight, socioeconomic factors, and weight gain during the first two years [6,7]. In our analysis, we first construct PIs for the children's BMI at approximately the age of four, relying on data available for the children at the age of two. In a second step, we explore the longitudinal structure of the present data and construct PIs for child-specific BMI patterns from two up to ten years.

Predicting child-specific BMI values is a great challenge from two different perspectives: From the epidemiological perspective, it is difficult to predict BMI values as they depend on factors which are hard to measure; such as physical activity, healthy nutrition, and lifestyle habits. From the statistical point of view, the distribution of BMI values is typically skewed and the degree of skewness depends on children's age, see e.g. Beyerlein et al. [8], which makes standard strategies to construct PIs relying on distributional and homoscedasticity assumptions problematic.

In these standard parametric approaches, first, a point prediction for the future BMI value is estimated based on mean regression models with Gaussian distributed errors, then a symmetric PI is constructed around that point based on distributional assumptions. To predict BMI values, however, these standard parametric approaches are problematic due to two reasons: not only the model assumptions for the point prediction might not be fulfilled but also the length of the PI depends on an assumed fixed variance which does not reflect the reality of an age-specific BMI skewness [9]. One possibility to overcome these problems would be the usage of more sophisticated parametric approaches, as for example generalized additive models for location scale and shape ("GAMLSS" [10]). GAMLSS are modelling up to four parameters of the conditional response's distribution and could therefore take age-specific skewness into account. This model class has already been used for constructing PIs in combination with boosting [11]. However, the construction of PIs based on GAMLSS depends totally on the assumed distribution and the interpretation of covariate effects with respect to the interval borders is not straightforward.

We avoid making distributional assumptions here by developing a new approach to constructing non-parametric prediction intervals based on quantile boosting. Instead of constructing intervals around a point prediction, the new approach directly models the interval borders by additive quantile regression [12]. The borders are fitted as BMI quantiles conditional on the child-specific covariate combination. We use quantile boosting for the estimation [13], which offers the advantage of flexible and inter-pretable covariate effects and an intrinsic variable selection property (which is in particular useful in high-dimensional data settings). The size of the resulting PIs is not fixed but depends on covariates - in longitudinal settings it might also depend on child-specific effects (corresponding to "random effects" in linear and additive mixed models).

During the work on this paper, we found a severe pitfall in the correct validation of prediction intervals. The appropriate measure for validating PIs is conditional coverage, not sample coverage (although being more intuitive) which makes it unfeasible in almost any data setting to evaluate the intervals in practice. The only way to demonstrate the correctness of PIs is therefore based on an empirical evaluation with simulated data. Thus, in a first step we evaluate the correctness of our approach in a set of simulation studies before applying quantile boosting to predict future BMI values.

## Methods

### Prediction intervals by conditional quantiles

The idea of using quantile regression to construct prediction intervals for new observations was presented in [12]. In contrast to standard regression analysis, quantile regression - thoroughly described in [14] - does not estimate the conditional expectation E(Y|X=x)=μ(x) of a random variable *Y *but the conditional quantile function *Q*_*τ*_(*Y*|*X *= *x*) = *q*_*τ*_(*x*) for a given *τ *∈ (0, 1) and a possible set of covariates *X *= *x*. Following the definition of quantiles as inverse of the cumulative distribution function, $q_{\tau }\left(x\right)=F_{Y|X=x}^{-1}\left(\tau \right)$, the probability of the response *Y *being smaller than *q*_*τ*_(*x*) is *τ*:

(1) $$P\left(Y<q_{\tau }\left(x\right)|X=x\right)=F_{Y|X}\left(q_{\tau }\left(x\right)\right)=\tau .$$

The goal is therefore to estimate the conditional quantile function ${\hat{q}}_{\tau }\left(x\right)$ by quantile regression based on a training sample (*y*_1_, *x*_1_), ..., (*y*_*n*_, *x*_*n*_). For a new observation, the specific covariate combination *x*_new _is plugged into ${\hat{q}}_{\tau }\left(x_{\text{new}}\right)$. A prediction interval for *y*_new _is then estimated by evaluating ${\hat{q}}_{\tau }\left(x_{\text{new}}\right)$ at $\tau_{1}=\frac{\alpha }{2}$ and $\tau_{2}=1-\frac{\alpha }{2}$, leading to

(2) $${\hat{\text{PI}}}_{\left(1-\alpha \right)}\left(x_{\text{new}}\right)=\left[\hat{q}\frac{\alpha }{2}\left(x_{\text{new}}\right),{\hat{q}}_{1}-\frac{\alpha }{2}\left(x_{\text{new}}\right)\right].$$

The resulting PI should cover a new observation *y*_new _with probability (1 - *α*) while its length depends on *x*_new_. There might be combinations of co-variates that allow for a very precise prediction for *y*_new _resulting in a narrow interval, whereas wide intervals imply that for a given *x*_new _the prediction is more inaccurate. As the estimates ${\hat{q}}_{\tau }\left(x\right)$ depend on a training sample (*y*_1_, *x*_1_), ..., (*y*_*n*_, *x*_*n*_), which are realizations of random variables *Y *and *X*, the boundaries of the intervals itself can be seen as random variables. This is an analogy to confidence intervals, which usually should cover unknown but fixed parameters. The boundaries of confidence intervals depend on the underlying sample and thus differ from sample to sample. Yet, for every sample, they cover the true parameter with a probability of 1 - *α*. Prediction intervals are constructed in the same way, but they cover a future realization of a random variable, which itself is random. The result is that the length of a prediction interval for *y*_new _is always larger than the length of a confidence interval for the expected mean of *Y*. Prediction intervals do not only take into account the sampling error made by the estimation based on a sample, but also the unexplained variability of *Y *given *X *= *x*. In conclusion, as long as *Y*|*X *= *x *is not deterministic, the length of the corresponding PI - in contrast to a confidence interval - does not reduce to 0, not even for infinitely large sample sizes.

### Conditional coverage vs. sample coverage

We stated that a correctly specified prediction interval PI_(1 - *α*)_(*x*_new_) covers a new observation *y*_new _with probability *π *: = (1 - *α*). To validate a method for fitting PIs, we obviously need a certain amount of new observations: From a single observation (*y*_new_, *x*_new_) it is impossible to verify if PI_(1 - *α*)_(*x*_new_) is correct. It either covers *y*_new _or not - both events do not prove anything, at least if *α *is not 0. Yet, if we have a certain amount of new observations, there still exist two different interpretations for the coverage probability *π*:

#### Sample coverage

For any new sample ***y ***= (*y*_1_,...,*y*_*n*_)^⊤ ^and corresponding covariates ***x ***= (*x*_1_, ...,*x*_*n*_)^⊤ ^about (1 - *α*) - 100% of the new sample ***y ***will be covered by the *n *prediction intervals PI(*x*_1_),...,PI(*x*_*n*_). The coverage refers to the whole sample. To evaluate sample coverage in practice, one estimates the coverage probability by averaging over different PIs:

(3) $$\hat{\pi }=\hat{E}\left(Y\in \text{PI}\left(x\right)\right)=\frac{\sum_{i=1}^{n}I\left\{y_{i}\in \text{PI}\left(x_{i}\right)\right\}}{n}$$

where *I*{·} is an indicator function.

#### Conditional coverage

For any *x*_new _and a corresponding sample (*y*_1_, *x*_new_),..., (*y*_*n*_, *x*_new_), about (1 - *α*) · 100% of the observations with the particular covariate combination *x*_new _will be covered by the prediction interval PI(*x*_new_). The coverage therefore refers to observations belonging to this *x*_*new*_. To evaluate conditional coverage in practice, one estimates the conditional coverage probability by averaging over different new observations for one PI:

(4) $$\begin{array}{ll} \hat{\pi }|x_{\text{new}} & =\hat{E}\left(Y\in \text{PI}\left(x_{\text{new}}\right)|X=x_{\text{new}}\right)\;\\ & =\frac{\sum_{i=1}^{n}I\left\{y_{i}\in \text{PI}\left(x_{\text{new}}\right)\right\}}{n}\;\\ & \; \end{array}$$

Although sample coverage is the more intuitive interpretation of PIs, it is obvious that conditional coverage reflects in a better way what we really expect from a PI. For example, after constructing a 95% PI for the BMI of a child at the age of four, given all information available from the child as a two-year-old, we particularly expect the future BMI of this child with its exact measures to be covered with a probability of 95%. In frequentistic language, the BMI of 95% of children with exactly the same measures should be covered by the interval. The coverage should hold for every child and every possible combination of covariates not only on average for all children.

**Figure 1:**
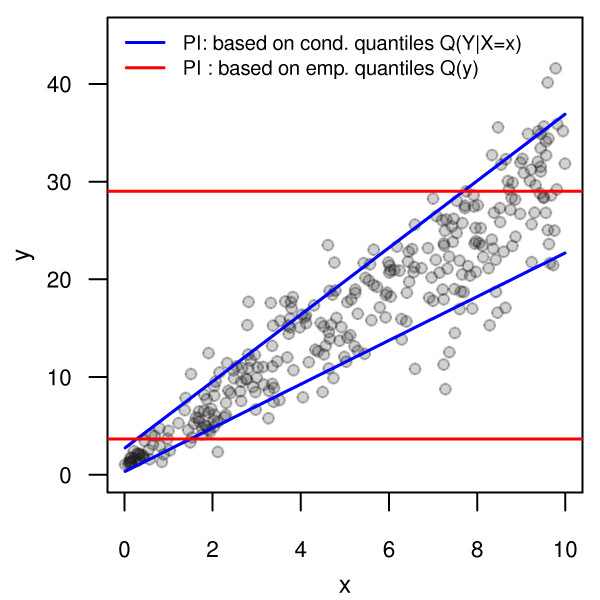
**Example to compare sample coverage and conditional coverage**. The blue lines represent a prediction interval for the response constructed by conditional quantiles. The red lines display a "naive" prediction interval constructed by the unconditioned empirical quantiles of the response in the training sample.

Hence, to show the correctness of PIs it is particularly not enough to show that PIs cover the right amount of observations on average from a new sample. This is further illustrated by a small example in Figure 1. For a simple univariate regression setting, two different prediction intervals were fitted: Both hold the sample coverage, but only one holds the conditional coverage. The first one, represented by the blue lines in Figure 1, relies on conditional quantiles fitted by linear quantile regression. It is an adequate interval for every possible *x*, it holds the conditional coverage and it adapts to the heteroscedasticity found in the data. The second one, drawn by red lines, is a "naive" interval, depending on the empirical quantiles of the response variable in the training sample. It does not take into account the information provided by *x *and is not adequate regarding the conditional coverage for any *x*. However, it holds the sample coverage. This further emphasizes the need to be aware of the different concepts of coverage probability and to clarify the precise aims of a PI analysis beforehand.

This finding leads to a severe problem, at least for multivariate prediction settings with several continuous covariates: For every combination of covariates only one response observation will be available under almost any practical circumstances. We will only find one child for each combination of covariates - not even twins will have the exact same measures - this is obviously not enough to verify the correct conditional coverage of a fitted PI.

Therefore, to demonstrate the correctness of a method fitting accurate prediction intervals, it is necessary to use artificial simulated data sets to evaluate the conditional coverage in (4) for a selected set of covariate combinations. Here, we will conduct a simulation study to evaluate if quantile boosting is a correct method to fit accurate conditional prediction intervals in potentially high-dimensional data settings before we apply this approach to predict future BMI values of children.

### Quantile boosting

Recall that our aim is to construct PIs based on conditional quantiles as given in (2). In our approach, we determine conditional quantiles by additive quantile regression. For a fixed quantile *τ *∈ (0,1), the conditional quantile function is expressed by an additive predictor as follows:

(5) $$q_{\tau }\left(x_{i}\right)=\eta_{\tau i}=\beta_{\tau 0}+\overset{p}{\underset{j=1}{\sum }}f_{\tau j}\left(x_{ij}\right).$$

The index *i *= 1, ...,*n*, denotes the individual, and *q*_*τ*_(***x***_*i*_) stands for the *τ*-quantile of the response *y*_*i *_conditional on its specific covariate vector ***x***_*i *_= (*x*_*i*1_, ..., *x*_*ip*_)^⊤^. The quantile-specific additive predictor *η*_*τi *_is composed of an intercept *β*_*τ*0 _and a sum of different effects of *p *covariates ***x***_*i *_= (*x*_*i*1_, ..., *x*_*ip*_)^⊤ ^on the quantile function. The functions *f*_*τ*1_, ..., *f*_*τp *_comprise linear effects, i.e. *f*_*τj*_(*x*_*ij*_) = *β*_*τj*_*x*_*ij*_, as well as non-linear effects whose functional form is not specified in advance. In fact, the additive predictor could also contain a wide variety of additional covariate effects, e.g. varying coefficient terms or spatial effects, as described in [13]. Note that contrary to classical regression, there is no specific distributional assumption for the response in (5). The only restriction is that the response must be continuous.

In general, the estimation of unknown parameters in quantile regression can be achieved by minimizing the empirical risk

(6) $${\hat{\eta }}_{\tau }=\underset{\eta_{\tau }}{\text{argmin}}\frac{1}{n}\overset{n}{\underset{i=1}{\sum }}\rho_{\tau }\left(y_{i},\eta_{\tau i}\right),$$

where the *check function ρ*_*τ *_is the appropriate loss function for fitting quantiles and can be written as:

(7) $$\rho_{\tau }\left(y,\eta_{\tau }\right)=\left\{\begin{array}{c} \tau \cdot \left|y-\eta_{\tau }\right|\;y>\eta_{\tau }\\ \left(1-\tau \right)\cdot \left|y-\eta_{\tau }\right|\;y\leq \eta_{\tau }. \end{array}\right)$$

Standard approaches for solving the optimization problem in (6) rely on linear programming [14,15]. Quantile regression forest [12] is a recent approach to conducting quantile regression and adapts random forest [16] to estimate the whole conditional distribution function. Since this approach is based on regression trees, the resulting estimates ${\hat{q}}_{\tau }\left(x\right)$ - in contrast to the additive modelling approach presented here - can only be described as black-box predictions. Nevertheless, we will use quantile regression forest as benchmark in our simulation study.

We will use gradient boosting for the estimation of the additive quantile regression model in (5), and call our approach *quantile boosting *in the following. Quantile boosting [13] was introduced as a method to flexibly estimate additive quantile regression models. It is an adaptation of component-wise functional gradient descent boosting [17] and aims at minimizing an empirical risk criterion, as given in (6). In case of quantile regression, the appropriate loss is the *check function *(7).

The minimization of (6) is achieved by stepwise updating the predictor function *η*_*τ*_. Therefore, *base-learners *are used, i.e. simple univariate regression models fitting the negative gradient of the empirical loss (7). The base-learners play a key role in the algorithm, since they define the kind of effects between each covariate and response. In our approach, we use simple linear models to represent linear covariate effects and penalized regression splines to represent non-linear effects. The advantage of quantile boosting is that the resulting predictor *η*_*τ *_is strictly additive and interpretable, following the additive quantile regression model in (5).

In detail, the boosting procedure works as follows: For each covariate, one specific base-learner is defined and in every boosting step the algorithm updates only the covariate with the best performing base-learner. This way, the algorithm is descending the loss by searching in the function space represented by the base-learners. If the algorithm is stopped before every base learner was at least once updated ("early stopping"), less important covariates will never have been updated during the boosting process and are effectively excluded from the final model. Thus, boosting comes along with an inherent variable selection property and produces sparse models in potentially high-dimensional settings. It even allows for candidate models that contain more covariates than observations.

Regarding prediction, early stopping is a desirable property, since it yields shrunk effect estimates. Shrinkage of effect estimates is a widely established method in statistical modelling [18,19] and tends to produce a more stable solution leading to an improved prediction accuracy of the model [20-22], even though an increase of the model bias (towards underlying data) has to be accepted. The primary aim is not to minimize the loss in the underlying training sample best - resulting in a small model bias - but to get accurate predictions with a small variance for new data. Since our work focuses on predictions for future BMI values, the shrinkage effect is of high relevance in our approach and is promising in order to provide accurate PIs.

A crucial parameter that has to be tuned with care during the boosting process is the number of stopping iterations. It should be tuned regarding the empirical loss in (6) on a test data sample, or - in case that no additional data is available - by applying cross-validation techniques or bootstrapping on the training data [19,23]. Quantile boosting is implemented within the R[24] add-on package **mboost **[25,26].

### Simulation study

We have already mentioned that the correct empirical validation of PIs should be based on conditional coverage. Since it is almost impossible to evaluate the conditional coverage in practical data analyses, we carried out a simulation study to provide some kind of proof that PIs fitted by quantile boosting are provided with correct conditional coverage. As benchmark, we used quantile regression forest [12] for which an implementation is available in the R add-on package **quantregForest **[12,27].

Our simulation study aims at answering the following questions:

1. Are the proposed PIs able to cover future observations with a predefined conditional coverage probability?

2. Is quantile boosting able to identify relevant informative covariates, also in high-dimensional settings, e.g. data sets with a potentially large number of covariates?

To investigate these questions, we generated artificial data from two different settings - a *linear setup *containing only covariates with linear effects on the response:

$$\begin{array}{c} Y_{i}=1.5-3x_{i1}-2x_{i2}+3x_{i3}+5x_{i4}+\overset{p}{\underset{j=5}{\sum }}0x_{ij}\\ \;+\left(1+.5x_{i1}+.5x_{i2}+.5x_{i3}+.5x_{i4}\right)\cdot \varepsilon_{i} \end{array}$$

and a *non-linear setup *including also non-linear effects:

$$\begin{array}{ll} Y_{i} & =2+3\text{sin}\left(\frac{2x_{i1}}{3}\right)+1.5\text{log}\left(x_{i2}\right)\;\\ & \;+2x_{i3}-2x_{i4}+\overset{p}{\underset{j=5}{\sum }}0x_{ij}\;\\ & \;+\left(0.7+1.5{\left(x_{i1}-1.5\right)}^{2}+.5\left(x_{i2}+x_{i3}\right)\right)\varepsilon_{i}.\;\\ & \; \end{array}$$

The first lines of the model formulas represent the contribution of the covariates *x*_1_, ..., *x*_*p *_on the expected mean of the response *y*, whereas the bottom line specifies their contribution to heteroscedasticity. Both settings include only four informative covariates *x*_1_,...,*x*_4_. The error terms *ε*_*i *_were drawn independent and identically from a standard normal distribution, i.e. *ε*_*i *_~ *N*(0,1), whereas the covariates were sampled independent and identically from a continuous uniform distribution, i.e. *x*_*i*1_,..., *x*_*ip *_~ *U*(0,1) for the linear setup and *x*_*i*1_, ...,*x*_*ip *_~ *U*(0, 3) for the non-linear setup. To evaluate the ability of quantile boosting to select relevant covariates, we generated data for both settings once in a low-dimensional scenario with *p *= 10 and once in a high-dimensional scenario with *p *= 500 which, in conclusion, included 496 non-informative covariates.

For each setting, we constructed two-sided 95% PIs

$${\hat{\text{PI}}}_{0.95}\left(x_{\text{new}}\right)=\left[{\hat{q}}_{0.025}\left(x_{\text{new}}\right),{\hat{q}}_{0.975}\left(x_{\text{new}}\right)\right]$$

in the following way: We generated in each simulation run a training sample (*y*_1_, ***x***_1_), ..., (*y*_*n*_, ***x***_*n*_), with *n *= 2000 observations and an additional data set with 5000 observations to select the optimal number of stopping iterations for quantile boosting. Then, we fitted additive quantile regression models and quantile regression forest for *τ*_1 _= 0.025 and *τ*_2 _= 0.975, including all *p *covariates.

In order to evaluate the conditional coverage of the resulting PIs, we pre-selected five fixed covari-ate combinations ***x***_*t *_with *t *= 1,..., 5, as test points and thereby tried to cover the *x*-space. For each of the five test points ***x***_*t*_, we sampled 10000 test observations *y*_test_|***x***_*t *_which served as "future" observations. In analogy to (4), we then estimated the conditional coverage of the resulting PIs separately for each model and test point by

$$\hat{\pi }|x_{t}=\frac{1}{10000}\overset{10000}{\underset{i=1}{\sum }}I\left\{y_{\text{test}i}\in {\hat{\text{PI}}}_{{}_{95\%}}\left(x_{t}\right)\right\}.$$

By designing our simulation in this way, we were able to evaluate the conditional coverage of the constructed PIs and avoided the pitfall of averaging over a new sample, corresponding to the sample coverage.

### Predicting childhood BMI

#### Data

Data contains observations from a prospective longitudinal birth cohort study (called "LISA study", [5]), including newborns between 11/1997 and 01/1999 from four German cities. Our aim is to predict future BMI values for children relying on the data available when they were two years old. Originally, the study included 3097 healthy children - of whom 2007 are complete cases in the sense that the necessary covariates at the age of two are all available for our analysis and at least one future BMI value until the age of ten is recorded. Continuous covariates from early childhood are the BMI of the child at birth (cBMI0) and as a two-year-old (cBMI2), the exact age of the child at the future measurement (cAge), the BMI of the mother at the beginning of pregnancy (mBMI) and the following BMI gain during pregnancy (mDiffBMI). The considered binary categorical covariates are the sex of the child (cSex), the area the child is living in (cArea - rural or urban), exclusive breastfeeding until the age of four months (cBreast), maternal smoking during pregnancy (mSmoke) and - with four covariate levels - the maternal level of education (mEdu - increasing by level). As potential response variables, the data comprises BMI values at approximately the age of four (cBMI4), six (cBMI6) and ten (cBMI10). See [9] for further description of the LISA study.

#### Cross-sectional analysis

The aim of our first analysis was to construct prediction intervals for future BMI values of individual children at approximately the age of four, relying on all information available from the child as a two-year-old. Therefore, we constructed two-sided 95% PIs with the following additive quantile regression model:

$$\begin{array}{ll} q_{\tau }\left(x_{i}\right) & =\beta_{\tau 0}+f_{\tau }\left(\text{cBMI}2_{i}\right)+\beta_{\tau 1}\text{cBMI}{\text{0}}_{i}\;\\ & \;+\beta_{\tau 2}\text{cAg}{\text{e}}_{i}+\beta_{\tau 3}\text{mBM}{\text{I}}_{i}+\beta_{\tau 4}\text{mDi}{\text{I}}_{i}\;\\ & \;+\beta_{\tau 5}\text{cSe}{\text{x}}_{i}+\beta_{\tau 6}\text{cAre}{\text{a}}_{i}+\beta_{\tau 7}\text{cBreas}{\text{t}}_{i}\;\\ & \;+\beta_{\tau 8}\text{mSmok}{\text{e}}_{i}+\beta_{\tau 9}\text{mEdu}2_{i}+\beta_{\tau 10}\text{mEdu}3_{i}\;\\ & \;+\beta_{\tau 11}\text{mEdu}4_{i}\;\\ & \; \end{array}$$

Here, *q*_*τ*_(***x***_*i*_) denotes the *τ*-quantile of the response cBMI4 for child *i *with covariate combination ***x***_*i*_. It will represent the borders of child-specific PIs for *τ*_1 _= 0.025 and *τ*_2 _= 0.975. We included a nonlinear effect for cBMI2 and linear effects for all other covariates in our candidate model.

As a benchmark, we compared PIs resulting from our approach to black box estimates for ${\hat{\text{PI}}}_{0.95}\left(x_{\text{new}}\right)$ from quantile regression forest. Yet, it was impossible to evaluate the conditional coverage of the PIs in our analysis as already discussed above. As a consequence, we focused on the empirical loss (6) for model comparison, which can be seen as a reliable measure not to validate but to compare algorithms fitting PIs by quantile regression. Thus, we determined the empirical loss for the two quantiles and both models in a 10-fold cross-validation analysis. The optimal stopping iteration for quantile boosting was selected by 25-fold bootstrapping on each of the 10 training data sets separately. Goodness-of-fit of the chosen models was assessed by a recent approach presented in Wei and He [28], originally developed for conditional growth charts. We generated test samples from the conditional model distribution and compared them to the observed empirical distribution of the response, see [28] for details.

#### Longitudinal analysis

In a second step, we tried to explore the longitudinal structure of the data at hand and constructed prediction intervals for BMI patterns of children until the age often, relying again on all information of the child as a two-year-old. As response, we now considered individual BMI values cBMI_*it *_for child *i *at three different time points *t *∈ {1,2,3} corresponding to the age of approximately four, six and ten. Note that related applications with similar longitudinal settings are the estimation of reference growth charts [29] and conditional growth charts [28]. We fitted the following additive quantile regression model for *τ*_1 _= 0.025 and *τ*_2 _= 0.975:

$$\begin{array}{ll} q_{\tau }\left(x_{it}\right) & =\beta_{\tau 0}+b_{\tau 1i}+b_{\tau 2i}\text{cAg}{\text{e}}_{it}+f_{1\tau }\left(\text{cAg}{\text{e}}_{it}\right)\;\\ & \;+f_{2\tau }\left(\text{cBMI}2_{i}\right)+\beta_{1\tau }\text{cBMI}{\text{0}}_{i}+\beta_{2\tau }\text{mBM}{\text{I}}_{i}\;\\ & \;+\beta_{3\tau }\text{mDi}{\text{I}}_{i}+\beta_{4\tau }\text{cSe}{\text{x}}_{i}+\beta_{5\tau }\text{cAre}{\text{a}}_{i}\;\\ & \;+\beta_{6\tau }\text{cBreas}{\text{t}}_{i}+\beta_{7\tau }\text{mSmok}{\text{e}}_{i}+\beta_{8\tau }\text{mEdu}2_{i}\;\\ & \;+\beta_{9\tau }\text{mEdu}3_{i}+\beta_{10\tau }\text{mEdu}4_{i}\;\\ & \; \end{array}$$

This model contains child and quantile specific intercept *b*_*τ*1*i *_and slope *b*_*τ*2*i *_to account for the correlation between repeated measurements of the same child, which typically occurs in longitudinal data. These individual-specific "random" effects are estimated by a specially designed base-learner employing *L*_1 _regularization methods [30]. In connection with *L*_1 _regularization, quantile regression for longitudinal data was first proposed by Koenker [31]. Here, we also include individual-specific slopes and smooth non-linear effects in the flexible predictor.

Contrary to the cross-sectional analysis, cAge is included and differs for different time points. The non-linear fixed effect *f*_1*τ *_describes an overall BMI pattern depending on age which is valid for all children, whereas the random effects *b*_*τ*2*i *_express child-specific linear deviations from this overall BMI pattern. All other covariates are time-constant. Again, we used the method presented in [28] to assess goodness-of-fit, in this case separately for the three different time points.

The optimal stopping iteration for the boosting algorithm was selected by applying subject-wise bootstrap. For this setting, it was impossible to compare quantile boosting to the benchmark algorithm, since quantile regression forest cannot account for a longitudinal data structure. Thus, we only calculated the PIs for BMI patterns of "new" children by ten-fold cross validation. To determine child-specfic PIs, for those children the child-specific intercepts and slopes were set to zero, which corresponds to their expected mean.

## Results

### Simulation study

Table 1 shows the resulting mean conditional coverage from 100 simulation runs for 95% PIs. Quantile boosting clearly outperforms quantile regression forest for both setups. Only for the borders of the *x-*space (***x***_4_, ***x***_5_) in the high-dimensional scenario, the PIs fail to cover 95% of "future" observations.

**Table 1:** Results simulation study

95% PIs	*p *= 10		*p *= 500	
	mboost	quantregForest	mboost	quantregForest
**Linear setup**				
$\hat{\pi }|x_{1}$	**0.9454**	0.9948	**0.9361**	0.9997
$\hat{\pi }|x_{2}$	**0.9489**	0.9689	**0.9425**	0.9889
$\hat{\pi }|x_{3}$	**0.9466**	0.9561	**0.9418**	0.9609
$\hat{\pi }|x_{4}$	**0.9437**	0.9307	0.9400	**0.9471**
$\hat{\pi }|x_{5}$	**0.9405**	0.9310	0.9373	**0.9534**

**Non-linear setup**				
$\hat{\pi }|x_{1}$	**0.9486**	0.9721	**0.9662**	0.9832
$\hat{\pi }|x_{2}$	**0.9494**	0.9925	**0.9623**	0.9961
$\hat{\pi }|x_{3}$	**0.9490**	0.9940	**0.9521**	0.9954
$\hat{\pi }|x_{4}$	**0.9460**	0.9785	**0.9407**	0.9792
$\hat{\pi }|x_{5}$	**0.9314**	0.8743	**0.9171**	0.8942

Mean conditional coverage resulting from 95% PIs for both setups and both scenarios. In every row, the value of the better performing algorithm (with the mean conditional coverage closer to the expected coverage of 95%) for each setup is printed in bold.

**Figure 2:**
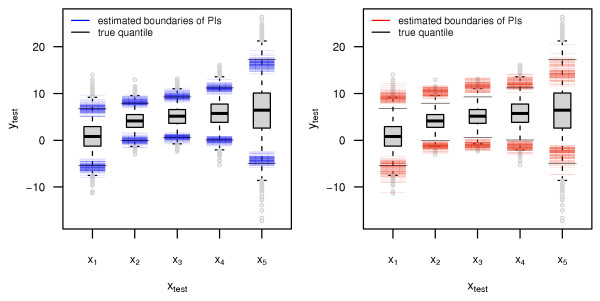
**Results from the non-linear setup in the low-dimensional scenario: Boxplots represent the distribution of *y***_**test**_. Black solid lines show the true conditional quantiles, while the colored lines represent the fitted conditional quantiles from 100 simulation runs for quantile boosting (left) and quantile regression forest (right).

Figure 2 further illustrates the concept of conditional coverage. The boxplots display the empirical distribution of the "future" observations for each of the test points ***x***_1_, ..., ***x***_5_. The solid black lines are the true conditional quantiles and represent the true borders of a 95% PI for each test point. The colored lines show the resulting estimated PI borders from 100 simulation runs of the two algorithms (quantile boosting on the left in blue, quantile regression forest on the right in red). As displayed by Figure 2 (non-linear setup, low-dimensional scenario), quantile boosting seems to work best in the center of the *x*-space, which is represented by test point ***x***_3_. For the other test points, the standard errors for the estimated quantiles get larger, yielding less accurate PIs. Quantile regression forest have more problems in fitting the correct conditional quantiles, which further explains why the resulting PIs fail to achieve the conditional coverage in Table 1.

**Figure 3:**
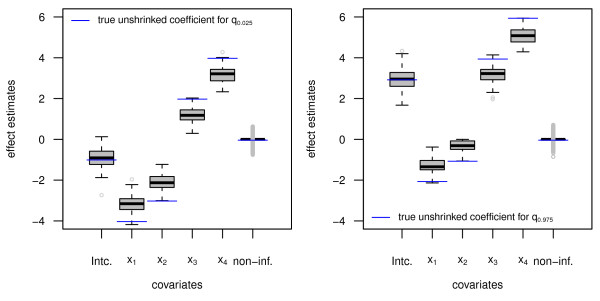
**Results from the linear setup, high-dimensional scenario, quantile boosting: Boxplots display the empirical distribution of the estimated coefficients for *q***_**0**:**025 **_**(left) and *q***_**0**:**975 **_**(right), obtained from 100 simulation runs**. The blue lines represent the underlying true coefficients without shrinkage.

Figure 3 displays the resulting effect estimates from 100 simulation runs of quantile boosting in the linear high-dimensional setup. The blue lines represent the quantile-specific true coefficients, which combine the effect of the covariate on the expected mean as well as on heteroscedasticity. The effect estimates corresponding to the non-informative covariates are combined in the rightmost boxplot. Therefore, Figure 3 illustrates the ability of the algorithm to select relevant covariates while intrinsically incorporating shrinkage of effect estimates.

In conclusion, PIs fitted by quantile boosting seem to cover future observations with the predefined coverage probability, conditional on the test points. The best results can be observed in the center of the *x*-grid. Quantile boosting outperforms the benchmark in both setups - linear and nonlinear setup - and for both scenarios - for the low-dimensional as well as for the high-dimensional scenario. However, the evaluated simulation setups did not include interaction terms - which could have favored quantile regression forest. For our data analysis, we can rely on the result that PIs constructed by quantile regression lead to correct conditional coverage probabilities. Furthermore, we can benefit from quantile boosting since the algorithm is able to select relevant covariates and yields sparse models in high-dimensional scenarios.

### Predicting childhood BMI

#### Data

**Figure 4:**
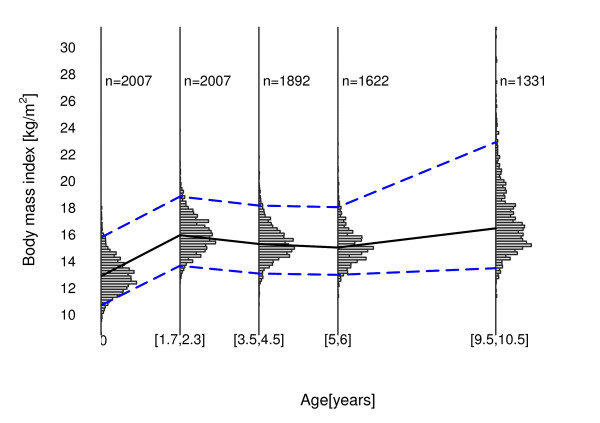
**Empirical BMI distribution in the LISA study depending on age**. The blue dashed lines represent the empirical quantile curves, i.e. *q*_0:025 _and *q*_0:975 _respectively, whereas the black solid line corresponds to the median.

To get a first impression of the data at hand, Figure 4 shows the empirical BMI distribution depending on age. It illustrates the age-specific skewness of the BMI distribution, beginning somewhere after the age of six, as well as the longitudinal data structure with repeated BMI observations per child at birth and around the age of 2, 4, 6, and 10.

#### Cross-sectional analysis

**Figure 5:**
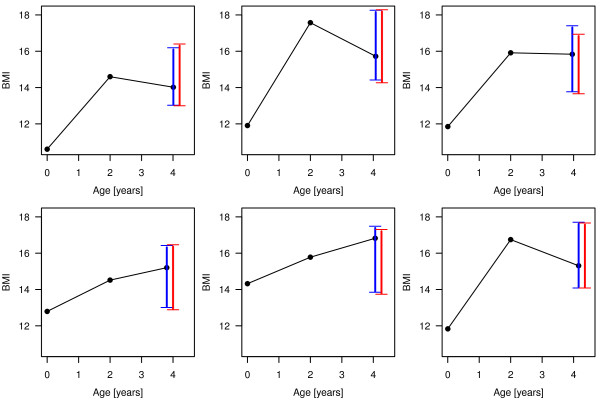
**Observed BMI patterns and resulting PIs from the cross-sectional analysis**. Blue intervals were fitted by quantile boosting, red ones by quantile regression forest. The six randomly selected children were part of the cases left out from the fitting process in the cross-validation analysis.

In our first cross-sectional analysis, we ignore the longitudinal character of the data and fit 95% PIs for the BMI around the age of four with data available from the children as two-year-olds, by both quantile boosting and quantile regression forest. Figure 5 shows the resulting PIs for six randomly chosen children (that were left out in the fitting process) emphasizing that length and level of the resulting PIs are in fact child-specific. The mean length of the PIs for all children is 3.55 kg/m^2^, while the lengths of the PIs range from 2.81 kg/m^2 ^to 5.62 kg/m^2^. Thus, the BMI prediction for some children is more precise than for others.

Table 2 contains the estimated covariate effects for quantile boosting. It can be observed that not all covariates are selected during the boosting process, which again reflects the variable selection property of quantile boosting. For the 2.5% BMI quan-tile, cSex, cBreast and mEdu are excluded from the model, whereas for the 97.5% BMI quantile cBMI0, mDiffBMI and cBreast are excluded. For both quantiles, mBMI and cArea are chosen with similar effects. This can be interpreted as follows with respect to the PIs: Both PI borders for a child from a urban area, for example, are shifted by -0.029 kg/m^2 ^and -0.075 kg/m^2 ^compared to the PI borders for a child from an rural area. Interestingly, the effect of mSmoke has different signs for different quantiles, meaning that the effect of maternal smoking during pregnancy seems to be negative for lower BMI quantiles and positive for upper BMI quantiles. This results in a wider PI for a child whose mother smoked during pregnancy. The estimated non-linear effects of cBMI2 are presented in the Additional file 1, Figure S1.

**Table 2:** Linear effect estimates for the LISA study: quantile boosting

	Cross-sectional analysis		Longitudinal analysis	
Variable	*τ *= 0.025	*τ *= 0.975	*τ *= 0.025	*τ *= 0.975
Intercept	14.208	14.867	14.627	12.723
cAge	--	--	*f*(·)	*f*(·)
cBMI2	*f*(·)	*f*(·)	*f*(·)	*f*(·)
cBMI0	0.008			
mBMI	0.028	0.034	0.029	0.132
mDiffBMI	0.026			
cSex = male		0.068		
cArea = urban	-0.029	-0.075		-0.043
cBreast = yes				
mSmoke = yes	-0.228	0.296		0.158
mEdu = 1 (low)		0.162		0.162
mEdu = 2		0.406		0.176
mEdu = 3		0.130		-0.107
mEdu = 4 (high)		0.070		-0.092

Resulting effect estimates for the borders of 95% PIs with the quantile boosting approach. Only effects of selected variables are displayed. Non-linear effect estimates are presented as Figure S1 and Figure S3 in Additional file 1.

**Figure 6:**
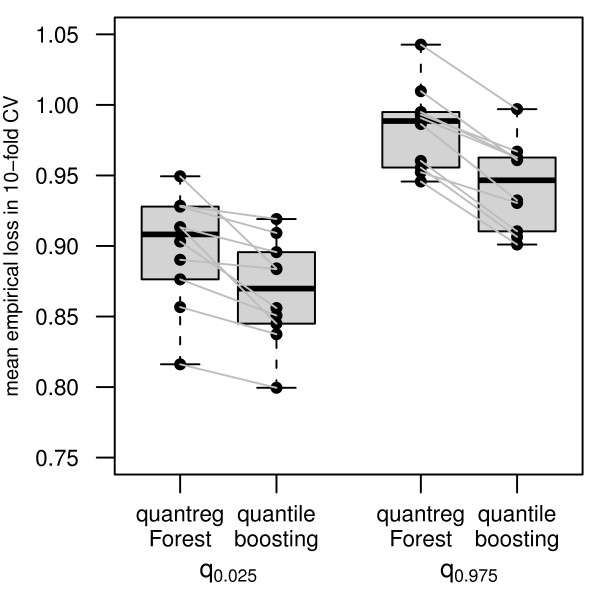
**Empirical loss distributions of the estimated quantiles on children iteratively left out in the fitting process in the 10-fold cross-validation**. Black points are the mean empirical loss from the 10 folds for every quantile and fitting method separately. Lower values indicate a more precise estimation of the corresponding quantile.

At first glance, the PIs resulting from quantile regression forest - the red-colored PIs in Figure 5 - seem to be very similar to those from quantile boosting: the mean length is 3.48 kg/m^2^, ranging from 2.46 kg/m^2 ^to 6.62 kg/m^2^. We also conducted a quantitative comparison between the algorithms by 10-fold cross-validation. Figure 6 displays the empirical loss distributions of the estimated quantiles on children iteratively left out in the fitting process. These results suggest that quantile boosting outperforms quantile regression forest with respect to accuracy in the estimation of the PI borders ${\hat{q}}_{0.025}\left(x_{\text{new}}\right)$ and ${\hat{q}}_{0.975}\left(x_{\text{new}}\right)$. This result is further supported by the goodness-of-fit diagnostic plots in the additional files (Additional File 1, Figure S2). The plots refer to the underlying models which were used to estimate the borders of the PIs and show a slightly improved goodness-of-fit for quantile boosting. Even though we cannot check the conditional coverage of our PIs here, we rely on the findings from the simulation study and conclude that quantile boosting does not only provide PIs with interpretable additive effects, but also yields more accurate predictions than quantile regression forest.

#### Longitudinal analysis

**Figure 7:**
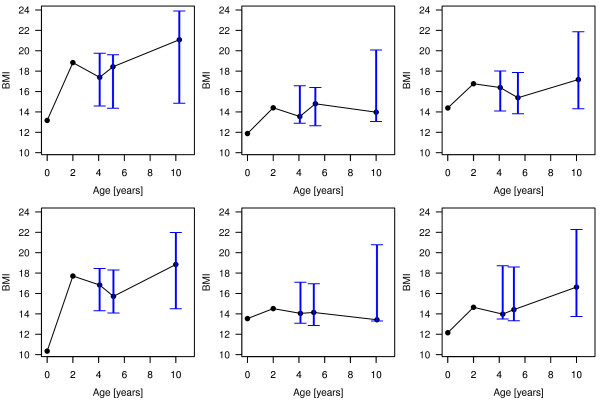
**BMI patterns and resulting PIs from the longitudinal analysis**. The six randomly selected children were part of the cases left out from the fitting process in the cross-validation analysis

In a second step, we used all information of the children at the age of two to predict their BMI patterns until the age of ten. Therefore, we included child-specific intercepts and slopes in the quantile boosting approach. Figure 7 shows the resulting PIs for six randomly chosen children.

Again, level and length of the PIs are child-specific, but the lengths of PIs at the age of ten are larger than the lengths at earlier time points. This seems to be realistic as we try to predict BMI values of children at the age of ten, only relying on information available as two-year-olds. The mean length of the PIs of all children is 4.78 kg/m^2^, ranging from 2.52 kg/m^2 ^to 11.28 kg/m^2^. The increased length of the intervals again results from the children getting older. This result is further emphasized by the estimated non-linear effects of cAge (presented as Figure S3 in the Additional file 1). The estimated effect for the 97.5% BMI quantile, i.e. the upper border of the PIs, is strongly increasing after the age of six, whereas the effect for the lower border remains constant. This result also corresponds to the empirical age-specific BMI distribution observed in Figure 4. Apparently the resulting PIs reflect the risk of childhood obesity kicking-in somewhere after the age of six.

Effect estimates for other covariates are included in Table 2. The pattern of selected covariates roughly corresponds to the cross-sectional analysis. Even though the effect signs and sizes show minor differences for some covariates, such as mEdu, the other effects on the PI borders remain stable across analyses, including the non-linear effect of cBMI2 (Additional file 1, Figure S3), confirming the presence of these effects. Diagnostic plots (Additional file 1, Figure S4) show a satisfying goodness-of-fit of the underlying models for the ages of four and six. Poorer results are obtained for the age of ten, which reflects the limited information available for this long-term prediction.

## Discussion

The aim of the present work was to construct prediction intervals for future BMI values of individual children. We pursued this aim by applying quantile boosting - a boosting approach estimating additive quantile regression models - to directly model the borders of the PIs. As a result, we do not rely on any distributional assumptions.

A main advantage of PIs fitted by quantile boosting is that we can directly interpret the estimated effects with regard to the interval borders. From the results of the cross-sectional analysis, for example, it follows that children whose mothers smoked during pregnancy have larger estimated PIs than other children. These conclusions could not have been drawn from quantile regression forest, an alternative approach to fitting non-parametric PIs, which leads to black box estimates.

The results of our simulation study suggest that quantile boosting outperforms quantile regression forest with respect to conditional coverage - which in our view is the key performance measure to evaluate PIs correctly. However, it is generally not possible to check conditional coverage in practical applications. In our data analyses, we thus had to rely on the findings from the simulation study. These findings were supported by the results of a formal comparison of empirical risks in the cross-sectional analysis, suggesting that quantile boosting provided more accurate predictions than quantile regression forest.

We could also benefit from the inherent shrinkage and variable selection properties of boosting in our analysis. Only a limited number of covariates was selected by the boosting algorithm, leading to sparse models. Note that it would even be possible to apply quantile boosting to data sets with more co-variates than observations, i.e., in high-dimensional data settings. A limitation coming along with the shrinkage property is the absence of standard errors estimations for the effect estimates. As a result, we cannot compute statistical tests regarding the effects of covariates, e.g. report information about their significance. Although researchers in practice often feel uncomfortable in the absence of p-values, we think that this limitation is acceptable here, as the focus is directed towards getting reliable predictions.

The resulting PIs of the longitudinal analysis emphasize further strengths of quantile boosting for fitting PIs. Relying on data available of the children as two-year-olds, we can fit accurate and child-specific PIs not only for BMI values around the age of four, but also for BMI patterns until the age of ten. Quantile boosting allows to explore longitudinal data structures by including individual-specific "random" effects, emphasizing the child-specific character for the resulting PIs. Here, we could observe that the lengths of the intervals strongly increase with the age of the children. From a methodological view, this absolutely reflects what we should expect from a valid method to fit PIs: The intervals do what they should, in reporting the increasing uncertainty in the prediction of BMI values until the age of ten based only on very limited information from the children in early childhood.

The lack of covariates explaining physical activity, nutrition and lifestyle habits of the children is of course a further limitation of the presented work. It would be interesting to see if this information could help for getting more precise predictions as presented in this paper.

## Conclusion

In conclusion, we think that quantile boosting is a promising approach to construct prediction intervals with correct conditional coverage in a non-parametric way. It can be applied to longitudinal settings and is therefore in particular suitable for the prediction of BMI patterns or similar data, where assumptions of standard parametric approaches are not fulfilled.

## Competing interests

The authors declare that they have no competing interests.

## Authors' contributions

All authors contributed to the conception of the manuscript and to the design of simulation study and data analyses. AM implemented the simulation study, carried out the data analyses and wrote the first draft of the manuscript, which was thoroughly revised by NF. All authors were involved in the final writing process and approved the final version.

## Pre-publication history

The pre-publication history for this paper can be accessed here:

http://www.biomedcentral.com/1471-2288/12/6/prepub

## Supplementary Material

Additional file 1 **Additional figures**. Document containing following additional figures not included in the main manuscript: *Figure S1 *Resulting estimates for the the non-linear partial effect of the BMI at the age of two on the PI for childhood BMI around the age of four. The lines represent the partial effect on *q*_0:025 _and *q*_0:975 _respectively as the borders of a 95% PI in the cross-sectional analysis. *Figure S*2 Goodness-of-fit diagnostic plots according to [28] for the underlying models from the cross-sectional analysis (BMI of children at the age of four). Test observations were simulated from the conditional model distribution and compared to the empirical distribution of the response observations (left plot). The right plot shows the standardized deviation of quantiles from the simulated conditional distribution to the real ones. Blue points and bars refer to the results of quantile boosting whereas red points and bars refer to those from quantile regression forest. *Figure S*3 Resulting estimates for the the non-linear partial effect of the BMI at the age of two (left) and the age of the child (right) on the PIs for childhood BMI patterns. The lines represent the partial effect on *q*_0:025 _and *q*_0:975 _respectively as the borders of a 95% PI in the longitudinal analysis. *Figure S4 *Goodness-of-fit diagnostic plots according to [28] for the underlying models from the longitudinal analysis (BMI of children at the ages of four, six and ten). Separately for the three different time points, test observations were simulated from the conditional model distribution and compared to the empirical distribution of the response observations in QQ-plots (first row). Barplots (second row) show the standardized deviation of quantiles from the simulated conditional distribution to the real ones. Click here for file

## References

[B1] Sassi F Devaux M Cecchini M Rusticelli E The Obesity Epidemic: Analysis of Past and Projected Future Trends in Selected OECD Countries OECD Health Working Papers 2009 45

[B2] Dehghan M Akhtar-Danesh N Merchant A Childhood Obesity, Prevalence and Prevention Nutrition Journal 2005 4 24 10.1186/1475-2891-4-24 16138930 PMC1208949

[B3] Jansen I Katzmarzykt P Srinivasan S Chenl W Malina R Bouchard C Berenson G Utility of Childhood BMI in the Prediction of Adulthood Disease: Comparison of National and International References Obesity Research 2005 13 1106 1115 10.1038/oby.2005.129 15976154

[B4] Whitaker R Wright J Pepe M Seidel K Dietz W Predicting Obesity in Young Adulthood from Childhood and Parental Obesity New England Journal of Medicine 1997 337 13 869 873 10.1056/NEJM199709253371301 9302300

[B5] LISA-plus Study Group 1998 Information about the study is available at http://www.helmholtz-muenchen.de/epi/arbeitsgruppen/umweltepidemiologie/projects-projekte/lisa-plus/index.html

[B6] Reilly JJ Armstrong J Dorosty AR Emmett PM Ness A Rogers I Steer C Sherriff A Early Life Risk Factors for Obesity in Childhood: Cohort Study British Medical Journal 2005 330 1357 1364 10.1136/bmj.38470.670903.E0 15908441 PMC558282

[B7] Beyerlein A Toschke AM von Kries R Risk Factors for Childhood Overweight: Shift of the Mean Body Mass Index and Shift of the Upper Percentiles: Results From a Cross-Sectional Study International Journal of Obesity 2010 34 4 642 648 10.1038/ijo.2009.301 20084072

[B8] Beyerlein A Fahrmeir L Mansmann U Toschke A Alternative Regression Models to Assess Increase in Childhood BMI BMC Medical Research Methodology 2008 8 59 10.1186/1471-2288-8-59 PMC2543035 18778466

[B9] Fenske N Fahrmeir L Rzehak P Höhle M Detection of Risk Factors for Obesity in Early Childhood with Quantile Regression Methods for Longitudinal Data Technical Report, Department of Statistics, University of Munich 2008 038

[B10] Rigby RA Stasinopoulos DM Generalized Additive Models for Location, Scale and Shape (with Discussion) Applied Statistics 2005 54 507 554 10.1111/j.1467-9876.2005.00510.x

[B11] Mayr A Fenske N Hofner B Kneib T Schmid M GAMLSS for High-Dimensional Data - a Flexible Approach Based on Boosting Journal of the Royal Statistical Society, Series C (Applied Statistics) 2012 [To appear]

[B12] Meinshausen N Quantile Regression Forests Journal Machine Learning Research 2006 7 983 999

[B13] Fenske N Kneib T Hothorn T Identifying Risk Factors for Severe Childhood Malnutrition by Boosting Additive Quantile Regression Journal of the American Statistical Association 2011 106 494 494 510 10.1198/jasa.2011.ap09272

[B14] Koenker R Quantile Regression 2005 New York: Cambridge University Press

[B15] Koenker R Ng P Portnoy S Quantile Smoothing Splines Biometrika 1994 81 4 673 680 10.1093/biomet/81.4.673

[B16] Breiman L Random Forests Machine Learning 2001 45 5 32 10.1023/A:1010933404324

[B17] Friedman JH Greedy Function Approximation: A Gradient Boosting Machine Annals of Statistics 2001 29 1189 1232

[B18] Tibshirani R Regression Shrinkage and Selection via the Lasso J Roy Statist Soc Ser B 1996 58 267 288

[B19] Bühlmann P Hothorn T Boosting Algorithms: Regularization, Prediction and Model Fitting Journal of Statistical Science 2007 22 4 477 505 10.1214/07-STS242

[B20] Efron B Biased Versus Unbiased Estimation Advances in Mathematics 1975 16 259 277 10.1016/0001-8708(75)90114-0

[B21] Copas JB Regression, Prediction and Shrinkage Royal Statistical Society, Series B 1983 45 311 354

[B22] Hastie T Tibshirani R Friedman J The Elements of Statistical Learning: Data Mining, Inference and Prediction 2009 2 Springer

[B23] Hastie T Comment: Boosting Algorithms: Regularization, Prediction and Model Fitting Journal of Statistical Science 2007 22 4 513 515 10.1214/07-STS242A

[B24] R Development Core Team R: A Language and Environment for Statistical Computing 2009 R Foundation for Statistical Computing, Vienna, Austria http://www.R-project.org. [ISBN 3-900051-07-0]

[B25] Hothorn T Bühlmann P Kneib T Schmid M Hofner Bmboost: Model-Based Boosting 2010 http://R-forge.R-project.org/projects/mboost. [R package version 2.1-0]

[B26] Hothorn T Bühlmann P Kneib T Schmid M Hofner B Model-based Boosting 2.0 Journal of Machine Learning Research 2010 11 2109 2113

[B27] Meinshausen N quantregForest: Quantile Regression Forests 2007 [R package version 0.2-2]

[B28] Wei Y He X Conditional Growth Charts Annals of Statistics 2006 34 2069 10.1214/009053606000000623

[B29] Wei Y Pere A Koenker R He X Quantile Regression Methods for Reference Growth Charts Statistics in Medicine 2006 25 8 1369 1382 10.1002/sim.2271 16143984

[B30] Kneib T Hothorn T Tutz G Variable Selection and Model Choice in Geoadditive Regression Models Biometrics 2009 65 2 626 634 [Including the web-based supplementary] 10.1111/j.1541-0420.2008.01112.x 18759832

[B31] Koenker R Quantile Regression for Longitudinal Data Journal of Multivariate Analysis 2004 91 74 89 10.1016/j.jmva.2004.05.006

