# Supplementary material for: Prediction intervals for future BMI values of individual children - a non-parametric approach by quantile boosting
Source: BMC Med Res Methodol. 2012 Jan 25;12:6. doi: 10.1186/1471-2288-12-6 (PMC3292459; doi:10.1186/1471-2288-12-6)
Supplement: Additional file 1 — Additional figures. Document containing following additional figures not included in the main manuscript: Figure S1 Resulting estimates for the the non-linear partial effect of the BMI at the age of two on the PI for childhood BMI around the age of four. The lines represent the partial effect on q0:025 and q0:975 respectively as the borders of a 95% PI in the cross-sectional analysis. Figure S2 Goodness-of-fit diagnostic plots according to [28] for the underlying models from the cross-sectional analysis (BMI of children at the age of four). Test observations were simulated from the conditional model distribution and compared to the empirical distribution of the response observations (left plot). The right plot shows the standardized deviation of quantiles from the simulated conditional distribution to the real ones. Blue points and bars refer to the results of quantile boosting whereas red points and bars refer to those from quantile regression forest. Figure S3 Resulting estimates for the the non-linear partial effect of the BMI at the age of two (left) and the age of the child (right) on the PIs for childhood BMI patterns. The lines represent the partial effect on q0:025 and q0:975 respectively as the borders of a 95% PI in the longitudinal analysis. Figure S4 Goodness-of-fit diagnostic plots according to [28] for the underlying models from the longitudinal analysis (BMI of children at the ages of four, six and ten). Separately for the three different time points, test observations were simulated from the conditional model distribution and compared to the empirical distribution of the response observations in QQ-plots (first row). Barplots (second row) show the standardized deviation of quantiles from the simulated conditional distribution to the real ones. [file 1471-2288-12-6-S1.PDF]

Additional figures

Prediction intervals for future BMI values of individual children – a non-parametric approach by quantile boosting

Figure S1

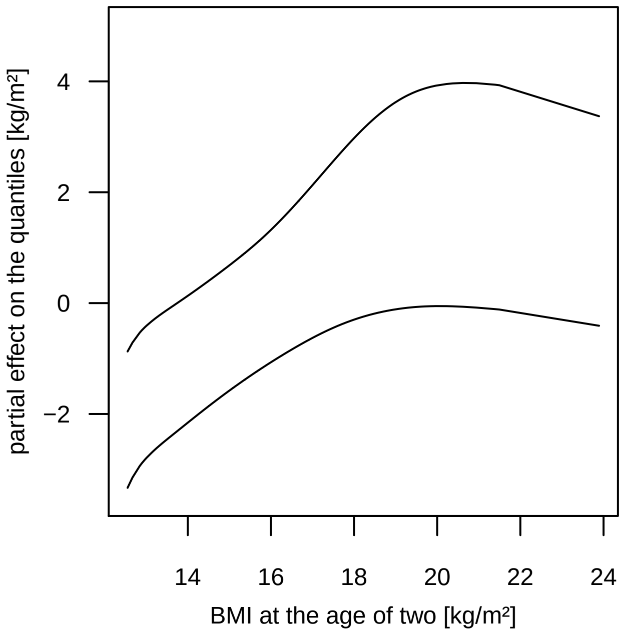

Figure A1: Resulting estimates for the the non-linear partial effect of the BMI at the age of two on the PI for childhood BMI around the age of four. The lines represent the partial effect on $q_{0.025}$ and $q_{0.975}$ respectively as the borders of a 95% PI in the cross-sectional analysis.

Figure S2

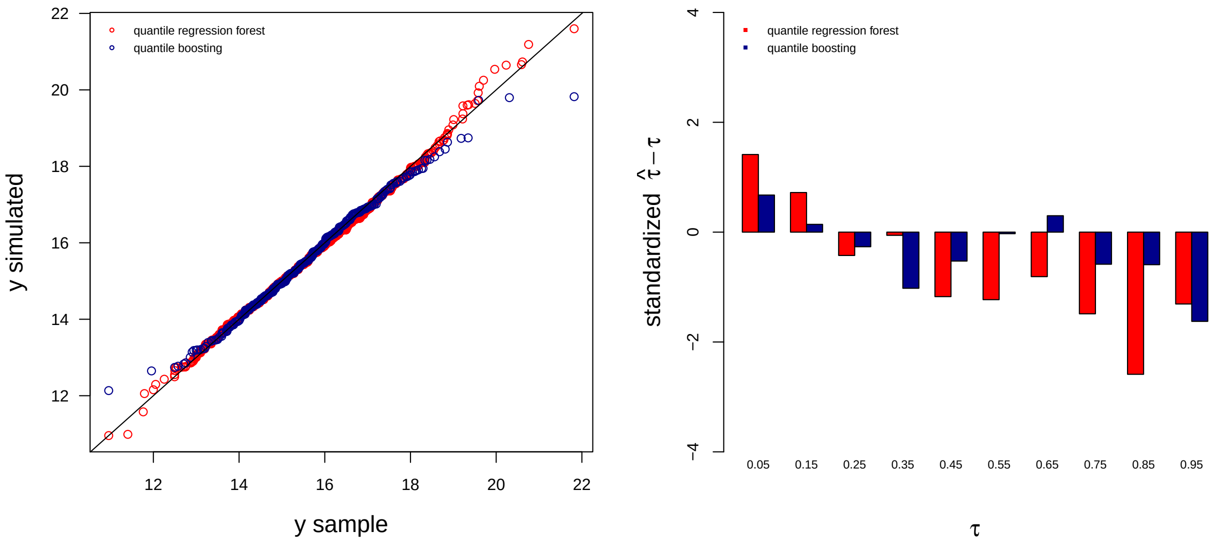

Figure A3: Goodness-of-fit diagnostic plots according to [28] for the underlying models from the cross-sectional analysis (BMI of children at the age of four). Test observations were simulated from the conditional model distribution and compared to the empirical distribution of the response observations (left plot). The right plot shows the standardized deviation of quantiles from the simulated conditional distribution to the real ones. Blue points and bars refer to the results of quantile boosting whereas red points and bars refer to those from quantile regression forest.

Figure S3

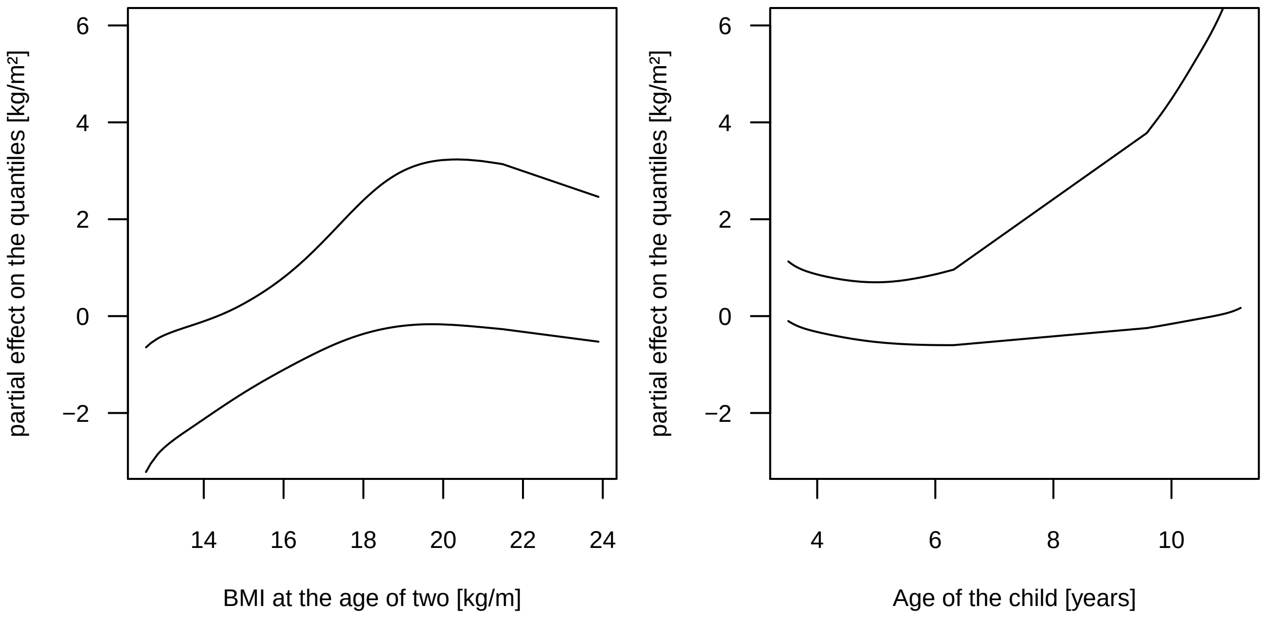

Figure A2: Resulting estimates for the the non-linear partial effect of the BMI at the age of two (left) and the age of the child (right) on the PIs for childhood BMI patterns. The lines represent the partial effect on $q_{0.025}$ and $q_{0.975}$ respectively as the borders of a 95% PI in the longitudinal analysis.

Figure S4

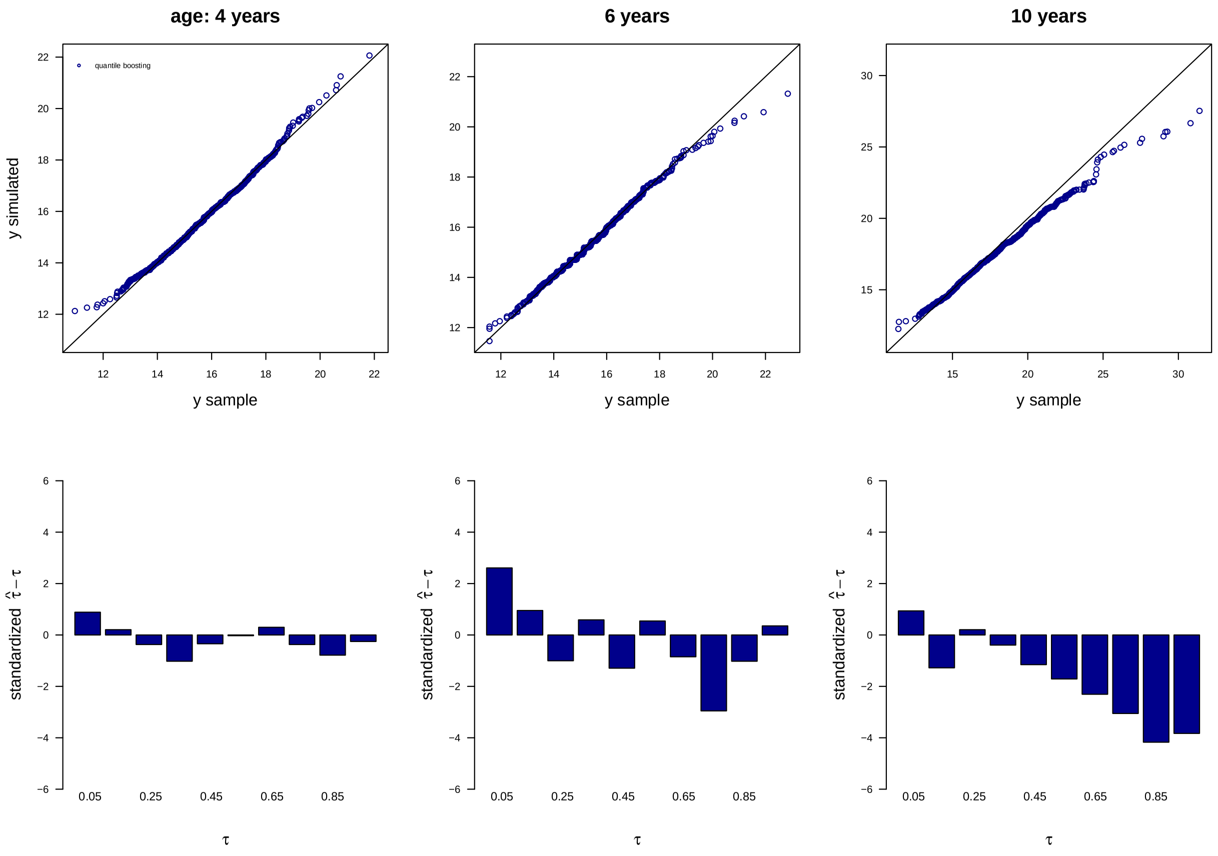

Figure A4: Goodness-of-fit diagnostic plots according to [28] for the underlying models from the longitudinal analysis (BMI of children at the ages of four, six and ten). Separately for the three different time points, test observations were simulated from the conditional model distribution and compared to the empirical distribution of the response observations in QQ-plots (first row). Barplots (second row) show the standardized deviation of quantiles from the simulated conditional distribution to the real ones.
